# Significance of the peripheral blood Treg/Th17 ratio as a prognostic immune biomarker in newly diagnosed multiple myeloma and its correlation with 1q21 gain/amplification

**DOI:** 10.3389/fimmu.2025.1595613

**Published:** 2025-05-29

**Authors:** Jingjing Wen, Qiaolin Zhou, Fang Xu, Jing Yue, Ya Zhang, Yiping Liu, Jing Su, Xiaogong Liang

**Affiliations:** Department of Hematology, Mianyang Central Hospital, School of Medicine, University of Electronic Science and Technology of China, National Health Commission Key Laboratory of Nuclear Technology Medical Transformation, Mianyang, Sichuan, China

**Keywords:** multiple myeloma, 1q21, MYC, Treg, Th17, immune function, prognosis

## Abstract

**Background:**

Regulatory T (Treg) and T helper 17 (Th17) cells play opposing roles in immune responses, and their balance critically regulates the multiple myeloma (MM) microenvironment. Despite advances in immunotherapy, current risk stratification lacks immune biomarkers.

**Methods:**

We collected the peripheral blood and bone marrow samples from MM patients to investigate the relationships among 1q21 gain/amplification, the Treg/Th17 ratio, and *MYC* gene abnormalities at diagnosis, remission, and relapse. Additionally, we evaluated the prognostic impact of the Treg/Th17 ratio.

**Results:**

A total of 130 newly diagnosed MM patients were enrolled, with 82 patients evaluated for 1q21 gain/amplification. During remission, patients with 1q21 gain/amplification had a significantly higher Treg/Th17 ratio (1.59 vs. 0.85, P = 0.042) and *MYC* expression levels (70.54% vs. 32.76%, P = 0.042) compared to those without 1q21 gain/amplification. Furthermore, patients with an elevated Treg/Th17 ratio (>0.7) during remission exhibited slightly higher *MYC* expression (45.70% vs. 30.60%) than those with lower ratios (P = 0.451). Patients achieving partial response or better exhibited significantly higher Th17 levels (3.34%, range: 0.19–10.80%) at diagnosis compared to those without remission (0.29%, range: 0–2.18%, P = 0.033). The group of elevated Treg/Th17 ratio (> 1.0) at diagnosis exhibited significantly shorter PFS compared to the reduced ratio (≤ 1.0) group (13.87 months vs. 30.67 months, P = 0.006). R2-ISS staging showed no significant impact on PFS (P = 0.236). By assigning scores to R2-ISS stages and elevated Treg/Th17 ratio at diagnosis, patients were stratified into low-risk (1–3 scores) and high-risk (4–5 scores) groups. High-risk patients exhibited significantly worse PFS compared to low-risk patients (P = 0.022). The combined model integrating R2-ISS staging and Treg/Th17 ratio achieved a concordance index(C-index) of 0.8, surpassing the C-index of R2-ISS staging alone (0.562), demonstrating better predictive performance.

**Conclusion:**

A potential mechanistic connection exists between 1q21 gain/amplification and immunosuppression, and the role of the *MYC* gene in this mechanism has garnered substantial interest. Patients with a higher Treg/Th17 ratio at diagnosis are more prone to relapse. The combination of R2-ISS staging and the Treg/Th17 ratio at diagnosis demonstrates stronger predictive ability for relapse.

## 1 Introduction

Multiple myeloma (MM) is the second most common hematologic malignancy. Immune function plays a key role in MM. Previous research suggests that MM recurrence is driven by dynamic interactions between tumor cells and the immune microenvironment, resulting in the immune system’s impaired ability to recognize and eliminate MM cells (1–3). Tumor immunity is intricately linked not only to the tumor microenvironment but also to systemic immune regulation mediated by peripheral immune cells (4).

During the pathogenesis and progression of MM, significant immune suppression is observed (5, 6). Researches indicate that CD4+ T cell subsets play a critical role in MM development (7–10). Based on cytokine profiles and functional characteristics, CD4+ T cells are categorized into four major subtypes: T helper (Th)1, Th2, regulatory T (Treg), and Th17 cells (11, 12). Regulatory T cells (Tregs), which regulate overall immune responses against tumor cells, also play a critical role in immune suppression and immune evasion by myeloma plasma cells (13). An expansion of Tregs in the peripheral blood (PB) is associated with poor survival and increased tumor burden in MM (14, 15). Th17 cells, a recently identified CD4+ T-cell subset, are actively involved in inflammatory and autoimmune responses (16, 17). Tregs can suppress the function of Th17 cells through the secretion of inhibitory cytokines (18). Given their opposing roles in immune regulation, Treg and Th17 cells maintain a delicate balance that ensures immune activation remains in a moderate state—neither hyperactive nor suppressed. Disruption of the Treg/Th17 balance can significantly contribute to immune dysfunction. It is likely that the equilibrium between Treg and Th17 cells is particularly crucial for sustaining the homeostasis of antitumor immunity (16). Some researchers propose that the Treg/Th17 ratio exhibits greater clinical relevance and prognostic utility in MM patients than isolated assessments of Treg or Th17 cells levels (19). The Treg/Th17 balance within the MM microenvironment is regarded as a key indicator of immunoregulatory control (20).

Additionally, the chromosomal aberration 1q21+ is the most common prognostic genetic abnormality, occurring in approximately 32–40% of newly diagnosed multiple myeloma (NDMM) patients (21–23). The prognostic significance of 1q21 amplification remains debated (24). Gains of 1q21 are thought to influence disease progression via a dosage effect of certain potential driver oncogenes located within the 1q21 amplicon, including CKS1B, MCL1, ADAR1, IL6R, ANP32E and others (21), and MCL1 can contribute to *MYC* activation (25). Translocations involving *MYC* (8q24) occur as a secondary abnormality and are generally late events in the natural history of myeloma (26). Therefore, 1q21 gain/amplification could result in further *MYC* amplification, with possible clinical implications. As it is well-known that the cross-talk between tumor cells and the tumor microenvironment (TME) facilitates the dissemination of diverse clonal populations, which are recognized as key drivers of MM progression (27, 28). Immune cells are one of the important components of TME (29).

To date, limited studies have examined the relationship between Treg/Th17 and 1q21 status in MM. It is necessary to find novel immune-related biomarkers to predict immunotherapy efficacy and recurrence risk, particularly in the era of MM immunotherapy. In this study, we investigated the association between Treg/Th17 cell ratio and both 1q21 gain/amplification status and *MYC* expression in MM. Furthermore, we assessed the prognostic value of the Treg/Th17 ratio for predicting first relapse in NDMM.

## 2 Patients and methods

### 2.1 Patients

A total of 130 NDMM patients admitted to Mianyang Central Hospital between June 2015 and August 2024 were enrolled in this study. All patients met the diagnostic criteria outlined in the International Myeloma Working Group (IMWG) guidelines (30). Clinical data collected included gender, age, lactate dehydrogenase (LDH) levels, β2-microglobulin (β2-MG) levels, and the proportions of peripheral blood (PB) Treg and Th17 cells within the CD4+ T cell population.

### 2.2 Treg and Th17

The following cell subtypes were analyzed in this study:Treg (CD3+CD4+CD25+FOXP3+) and Th17 (CD3+CD4+IL-17+) cells (31, 32). Flow cytometry was used to investigate the frequencies of Treg and Th17 cells.

For Th17 cells analysis, 250 μL of heparin-anticoagulated PB samples was diluted 1:1 with RPMI 1640 culture medium (Hyclone, catolog: SH30027.01). A 1 μL of 500× cell stimulator (Thermo Fisher Scientific, catalog 00-4975-93) was added and thoroughly mixed. The mixture was transferred to a 6-well plate and incubated at 37°C with 5% CO_2_ for 5 hours. After incubation, red blood cells were lysed using a lysis buffer (Beyotime, catolog C3702). Cells were then harvested, washed, and stained for surface markers CD45 PerCP (catalog 045-104-3/clone 4A/Biotech), CD3 FITC (catalog 340542/clone SK7/BD), and CD4-PEcy7 (catalog 348799/clone SK3/BD). Intracellular cytokine staining was performed using the Intracellular Fixation & Permeabilization Buffer Set (eBiosciences, catalog 88-8824-00) according to the manufacturer’s instructions. Briefly, cells were fixed, permeabilized, and incubated with IL-17A PE (catalog 12-7179-42/clone eBio64 DEC 17/Thermo Fisher) for 30–40 minutes at room temperature in the dark. The percentage of IL-17A+ cells was calculated relative to the total CD3+CD4+ T cell population.

For Tregs analysis, red blood cells in whole blood samples were lysed, followed by surface staining for CD45 PerCP (catalog 045-104-3/clone 4A/Biotech), CD3 FITC (catalog 340542/clone SK7/BD), CD4-PEcy7 (catalog 348799/clone SK3/BD), and CD25 APC (catalog 567316/clone BC96/BD). Nuclear transcription factor FOXP3 staining was performed using the FOXP3/Transcription Factor Staining Buffer Set (eBiosciences, catalog 00-5523-00) according to the manufacturer’s instructions. Cells were fixed, permeabilized, and incubated with FOXP3 PE (catalog 12-4776-42/clone PCH101/Thermo Fisher) for 60 minutes at room temperature in the dark.

**Figure 1:**
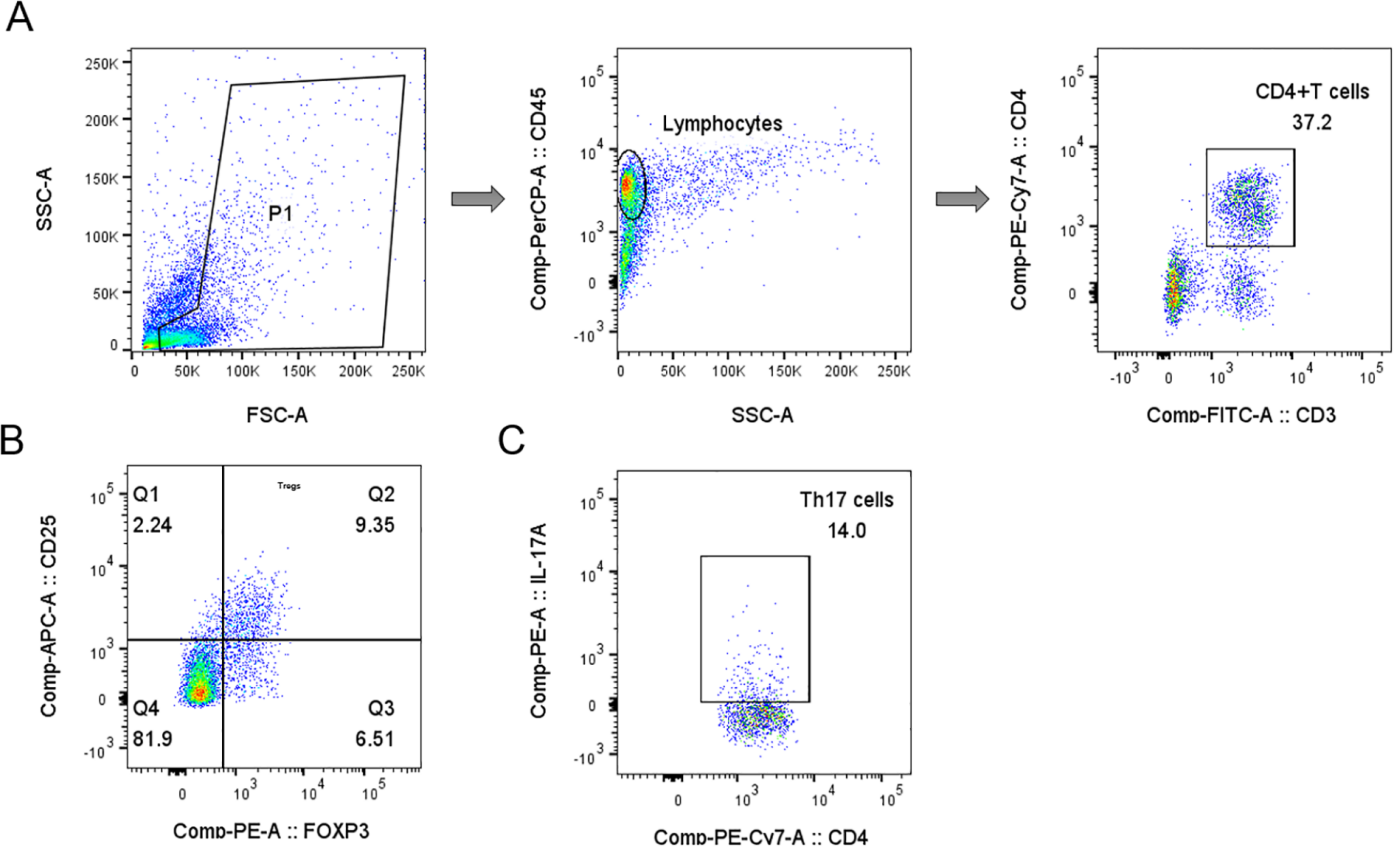
Gating strategy for Tregs and Th17 cells analyses in representative samples of peripheral blood mononuclear cells. **(A)** Schematic diagram of the flow cytometry analyses of CD3+ CD4+ T cells. **(B)** Q2 gate represents the percentage of Tregs (CD25+FOXP3+) among total CD4+ T cells. **(C)** The plot showing the gate used to identify the percentage of Th17 cells (CD4+IL-17+) in CD4+ T cells.

The proportions of Treg and Th17 cells were calculated as percentages of the total CD3+CD4+ T cell population. Samples were analyzed using a BD FACSCanto flow cytometer (BD Biosciences), and data were processed using DIVA software. The analysis of Treg and Th17 subsets was performed sequentially, as illustrated in **Figure 1**. Detection of Treg and Th17 cells was conducted by Sichuan West China Kang Shengda Medical Testing Co., Ltd.

### 2.3 MM-related cytogenetics analyses

Fluorescence *in situ* hybridization (FISH) combined with immunofluorescence was used for cytogenetic analysis. All bone marrow (BM) samples were enriched for plasma cells using CD138 immunomagnetic bead sorting. Genetic abnormalities were evaluated according to the positive thresholds established by the European Myeloma Network (33). Specifically, 1q21 gain/amplification was defined as ≥20% of nuclei displaying at least three copies of 1q21. The del(17p) and del(13q) were defined as ≥20% of nuclei showing deletions at 17p13 and 13q14.3, respectively. Translocations, including t(11;14)(q13;q32), t(4;14)(p16;q32), t(6;14)(p21;q32), and t(14;16)(q32;q23), were defined as ≥10% of nuclei exhibiting the respective translocation patterns. FISH analysis for MM was conducted by Sichuan West China Kang Shengda Medical Testing Co., Ltd.

### 2.4 Detection of *MYC* gene abnormalities

This study performed comprehensive molecular characterization of *MYC* gene in PB samples, including expression analysis, mutational screening and rearrangement detection. *MYC* gene expression levels were quantified using SYBR Green-based real-time quantitative PCR (RT-qPCR), with ABL1 serving as the endogenous reference gene for normalization. Absolute quantification was achieved through standard curve method to determine *MYC* and *ABL1* copy numbers, with *MYC*/*ABL1* ratio (expressed as percentage) calculated for expression normalization (Forward primer: 5’- CTCTCCGTCCTCGGATTCTC -3’; Reverse primer: 5’- ATCTTCTTGTTCCTCCTCAGAGT -3’). *MYC* gene rearrangements were identified by FISH targeting 8q24.21 locus. For mutation analysis, the entire *MYC* coding region and adjacent splice sites were examined by Sanger sequencing following PCR amplification, utilizing 7 PCR reactions and 14 sequencing runs to ensure complete coverage. Appropriate positive and negative controls were included throughout all experimental procedures to ensure data reliability. Detection of *MYC* gene was conducted by Sichuan West China Kang Shengda Medical Testing Co., Ltd.

### 2.5 Staging and risk stratification

Staging was performed using the Revised International Staging System (R-ISS) (34), Mayo Stratification of Myeloma and Risk-adapted Therapy (mSMART) (35), and the second revision of the International Staging System (R2-ISS) (36). For R2-ISS, baseline risk factors, including ISS stage, LDH elevation, del(17p), t(4;14), and 1q21+, were assigned weighted scores. Patients were stratified into four risk groups based on R2-ISS scores: low risk (R2-ISS I, 0 points), low-intermediate risk (R2-ISS II, 0.5–1 points), intermediate-high risk (R2-ISS III, 1.5–2.5 points), and high risk (R2-ISS IV, 3–5 points).

### 2.6 Treatment regimen and efficacy evaluation

All patients were treated in accordance with the guidelines for the diagnosis and management of MM (37, 38). Final treatment plans were established by consensus between clinicians and the patient. Treatment regimens were categorized according to induction and consolidation strategies. Specifically, 67 patients received bortezomib-based therapy, 3 patients underwent immunomodulatory drug-based treatment, 56 patients were administered a combination of bortezomib and immunomodulators, and 4 patients were treated with daratumumab-based regimens. Treatment efficacy was evaluated based on the 2016 IMWG criteria (39), which categorize responses into strict complete response (sCR), complete response (CR), very good partial response (VGPR), partial response (PR), minimal response (MR), stable disease (SD), and progressive disease (PD). Some patients underwent autologous hematopoietic stem cell transplantation (ASCT) after achieving at least a PR. Patients who responded effectively to induction therapy continued with consolidation and maintenance therapy until disease relapse, progression, or the occurrence of severe adverse drug reactions requiring a change in treatment. Maintenance therapy predominantly involved the use of proteasome inhibitors (PIs) or immunomodulatory drugs (IMiDs). The median follow-up time was 27.6 months (range: 0.4–111.7 months). Progression-free survival (PFS) was defined as the time from diagnosis to relapse, death, or the end of follow-up. Overall survival (OS) was defined as the time from diagnosis to death from any cause or the end of follow-up.

### 2.7 Statistical analyses

All statistical analyses were performed using SPSS version 26.0, while GraphPad Prism 8.0 was used to visualize the summarized data. Normality was assessed using Shapiro-Wilk tests. Normally distributed continuous data were expressed as mean ± standard deviation and analyzed using the t-test. Non-normally distributed data were presented as median (range) and compared using the Mann–Whitney U test. The Kruskal–Wallis H test was used for comparisons among the three groups. Categorical variables were evaluated by Chi-square test or Fisher’s exact test, as appropriate. Receiver operating characteristic (ROC) curves were used to determine the optimal cutoff value. Univariate survival analyses were carried out using the Kaplan Meir methods. The prognostic performance of the combined R2-ISS/Treg/Th17 risk stratification model was quantitatively assessed using the concordance index (C-index), with calculation performed using R statistical software version 4.2.1. The C-index value of 1.0 indicates perfect prediction 0.5 indicates no predictive ability. A two-tailed *p*-value < 0.05 was considered statistically significant. When performing pairwise comparisons among the three groups, a P-value < 0.0167 (Bonferroni correction) was considered statistically significant.

## 3 Results

### 3.1 Patients characteristics

A total of 130 NDMM patients admitted to Mianyang Central Hospital between June 2015 and August 2024 were enrolled in this study. Of these, 79 (60.8%) were male and 51 (39.2%) were female, with a median age of 63 years (range: 38–87 years). PFS was evaluable in 94 patients, and OS was evaluable in 96 patients. First relapse occurred in 39.4% of patients, with a median PFS of 30.7 months (95% CI: 26.58–34.82 months). The mortality rate was 9.4%, and the median OS was not reached. The 5-year survival rate was 68%. Among 85 patients assessable for R-ISS staging, 10.6% (9/85) were classified as stage I, 69.4% (59/85) as stage II, and 20.0% (17/85) as stage III. Among 81 patients assessable for R2-ISS staging, 9.9% (8/81) were classified as low risk, 11.1% (9/81) as low-intermediate risk, 59.3% (48/81) as intermediate-high risk, and 19.8% (16/81) as high risk. According to the Mayo mSMART 3.0 risk stratification, 38.5% of patients were classified as standard risk and 61.5% as high risk.

### 3.2 Clinical characteristics of patients with 1q21 gain/amplification

In the study involving 82 patients subjected to 1q21 testing, 57.3% (47 cases) manifested 1q21 gain/amplification. The distribution of ISS and R-ISS stages differed significantly between patients with 1q21 gain/amplification and those without 1q21 gain/amplification (*p < 0.05*). Specifically, pairwise comparisons demonstrated that a significantly higher proportion of patients with 1q21 gain/amplification were classified as ISS stage II and R-ISS stage III compared to those without 1q21 gain/amplification (*p* < 0.0167), **Table 1**.

**Table 1:** Comparison of clinical characteristics and survival times between patients with and without 1q21 gain/amplification.

Clinical Characteristics	Without 1q21 gain/amplification	With 1q21 gain/amplification	Statistical Value	*p*-value
Age	63.29 ± 12.16	63.49 ± 9.66	0.085	0.933
Gender			0.011	0.915
male	22 (62.9%)	29 (61.7%)		
female	13 (37.1%)	18 (38.3%)		
Platelet/Lymphocyte Ratio	183.61 (68.67~2613.86)	159.79 (33.85~2313.61)	-1.720	0.085
Lymphocyte/Monocyte Ratio	0.56 (0.09~3.16)	0.97 (0.12~2.30)	1.181	0.238
LDH (U/L)	184 (102~568)	172 (82~916)	1.561	0.119
Serum M-Protein (g)	26.21 ± 23.52	32.35 ± 22.72	1.074	0.287
ISS stage			6.505	0.039*
I	12 (34.3%)	7 (14.9%)		
II	8 (22.9%)	22 (46.8%)		
III	15 (42.9%)	18 (38.3%)		
R-ISS stage			12.843	0.001*
I	6 (19.4%)	1 (2.2%)		
II	24 (77.4%)	31 (68.9%)		
III	1 (3.2%)	13 (28.9%)		
With t (4:14)	1 (2.9%)	16 (34.8%)	12.217	0.000*
With t (14:16)	0 (0.0%)	3 (6.5%)	/	0.255
del (17p) or p53 mutations	5 (14.3%)	14 (30.4%)	2.887	0.089
With one of the high-risk cytogenetic abnormalities ^#^	6 (17.1%)	26 (57.8%)	13.545	0.000*
Treg at Diagnosis (%)	1.39 (0~6.01)	2.30 (1.07~9.41)	1.478	0.139
Treg at Remission (%)	4.83 ± 1.64	5.07 ± 1.63	0.225	0.828
Th17 at Diagnosis (%)	3.93 ± 2.28	4.96 ± 2.76	0.984	0.335
Th17 at Remission (%)	6.46 ± 3.37	3.53 ± 1.47	1.603	0.153
Treg/Th17 at Diagnosis	0.66 (0~5.7)	0.50 (0.29~3.18)	0.543	0.587
Treg/Th17 at Remission	0.85 ± 0.34	1.59 ± 0.56	2.478	0.042*
With *MYC* gene mutation at Diagnosis	33.3% (1/3)	84.6% (11/13)	NA	1.136
*MYC*/*ABL1*% at Diagnosis	65.41 (46.34~2384.14) %	79.45 (20.59~2011.50) %	0.062	0.950
*MYC*/*ABL1*% at Remission	32.76 ± 21.20 %	70.54 ± 32.62 %	2.186	0.042*

LDH, lactate dehydrogenase; ISS, International Staging System; R-ISS, Revised International Staging System; Treg, regulatory T; Th17, T helper (Th) 17. ^#^Patients had at least one of the following high-risk cytogenetic abnormalities, including del (17p), t (4;14), t (14;16), or p53 mutations. NA, Not applicable. *p < 0.05 was considered statistically significant.

The proportion of patients with 1q21 gain/amplification who also harbored t(4;14) was significantly higher compared to those without 1q21 gain/amplification (34.8% vs. 2.9%, *p* = 0.000). However, no significant differences were observed between patients with and without 1q21 gain/amplification in the occurrence of other high-risk cytogenetic abnormalities, including t(14;16), del(17p), or p53 mutations (all *p* > 0.05). Importantly, the proportion of patients with 1q21 gain/amplification harboring at least one high-risk cytogenetic abnormality—such as del(17p), t(4;14), t(14;16), or p53 mutations—was markedly higher than that of patients without 1q21 gain/amplification (57.8% vs. 17.1%, *p* =0.000), as summarized in **Table 1**.

### 3.3 Analysis of 1q21 gain/amplification in relation to Tregs and Th17 cells levels

**Figure 2:**
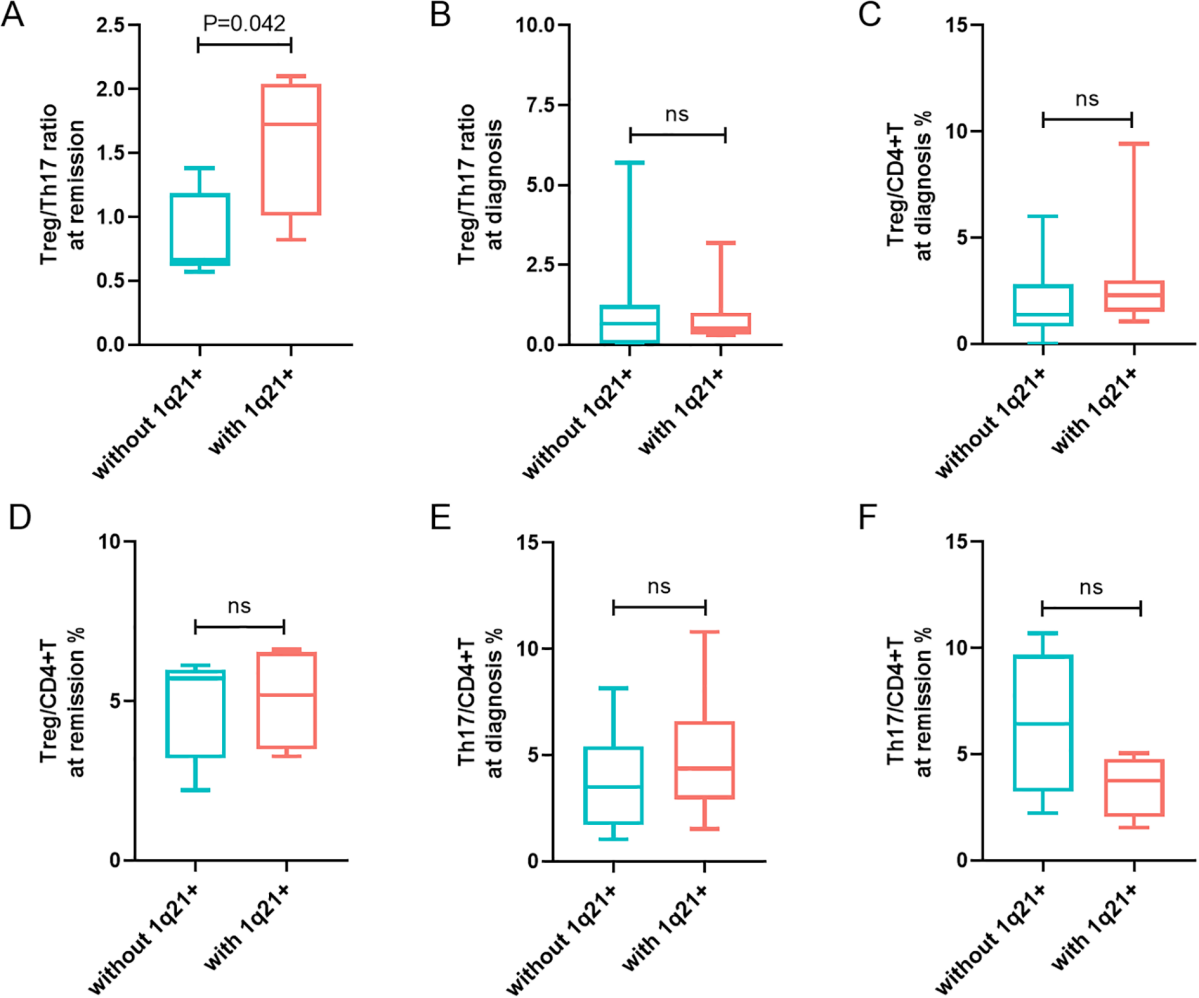
Analysis of 1q21 gain/amplification in relation to Tregs and Th17 cells levels. **(A, B)** Treg/Th17 ratio at remission and at diagnosis in patients with and without 1q21 gain/amplification. **(C, D)** Percentages of Tregs in CD4+T cells at diagnosis and at remission in patients with and without 1q21 gain/amplification. **(E, F)** Percentages of Th17 cells in CD4+T cells at diagnosis and at remission in patients with and without 1q21 gain/amplification. “ns”, represent not significant. *p* < 0.05 was considered statistically significant.

The median Treg/Th17 ratio during remission in patients with 1q21 gain/amplification at initial diagnosis was 1.59 ± 0.56%, significantly higher than that in patients without 1q21 gain/amplification (0.85 ± 0.34%), with a *p*-value of 0.042 (**Figure 2A**). However, there was no significant difference in the Treg/Th17 ratio at diagnosis between the groups with and without 1q21 gain/amplification (*p* > 0.05; **Figure 2B**), **Table 1**.

Patients with 1q21 gain/amplification at diagnosis exhibited slightly higher Treg percentages at both diagnosis and remission compared to those without 1q21 gain/amplification, although the differences were not statistically significant (*p* > 0.05; **Figures 2C, D**). Additionally, no significant differences in Th17 percentages were observed at diagnosis or remission between patients with and without 1q21 gain/amplification (*p* > 0.05; **Figures 2E, F**), **Table 1**.

**Figure 3:**
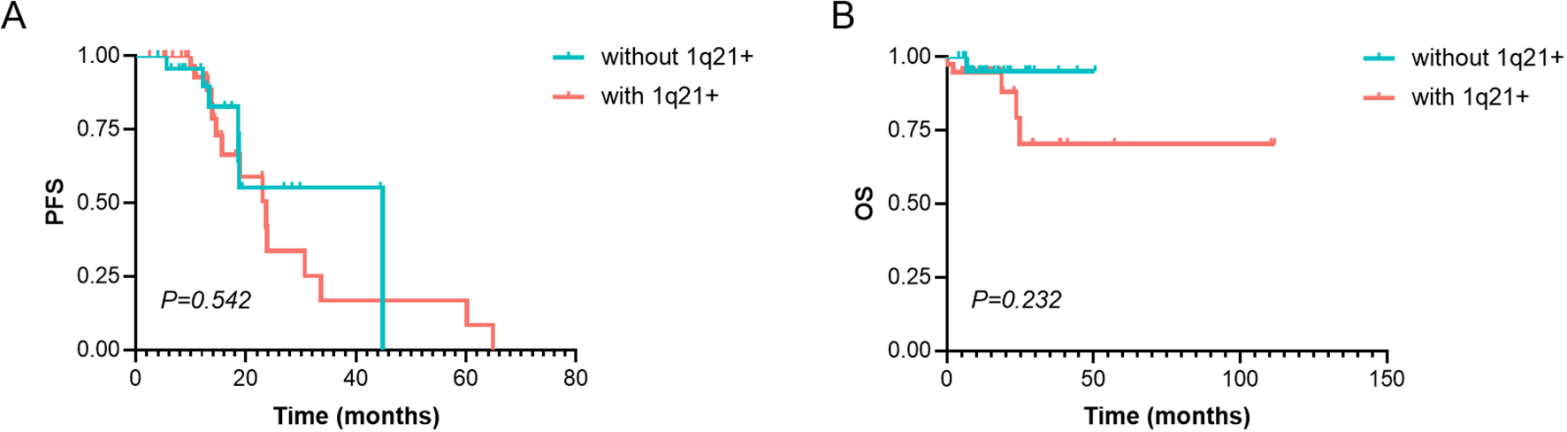
Survival analysis of PFS and OS in MM patients with or without 1q21 gain/amplification. **(A)** PFS and **(B)** OS analysis in patients with and without 1q21 gain/amplification. PFS, Progression-Free Survival. OS, Overall Survival. MM, Multiple Myeloma. *p* < 0.05 was considered statistically significant.

The median PFS in patients with 1q21 gain/amplification was 23.70 months, slightly shorter than that in patients without 1q21 gain/amplification (44.83 months), although the difference was not statistically significant (χ² = 0.371, *p* = 0.542; **Figure 3A**). Similarly, 1q21 gain/amplification status had no significant impact on OS (χ² = 1.430, *p* = 0.232; **Figure 3B**).

### 3.4 *MYC* expression in relation to 1q21 and the Treg/Th17 ratio

To investigate potential associations between 1q21 abnormalities and Treg/Th17 immune regulation, we performed comprehensive *MYC* gene profiling of PB samples from MM patients. This included analysis of *MYC* rearrangements (by FISH), mutation screening (via sanger sequencing), and quantitative expression assessment (using RT-qPCR).

In our cohort of 17 newly diagnosed patients who underwent *MYC* rearrangement testing: one patient (5.9%) showed *MYC* rearrangement negativity with increased gene copy number; one patient (5.9%) demonstrated concurrent *MYC* rearrangement and 1q21 gain/amplification, and the remaining 15 patients (88.2%) showed no *MYC* structural abnormalities.

**Figure 4:**
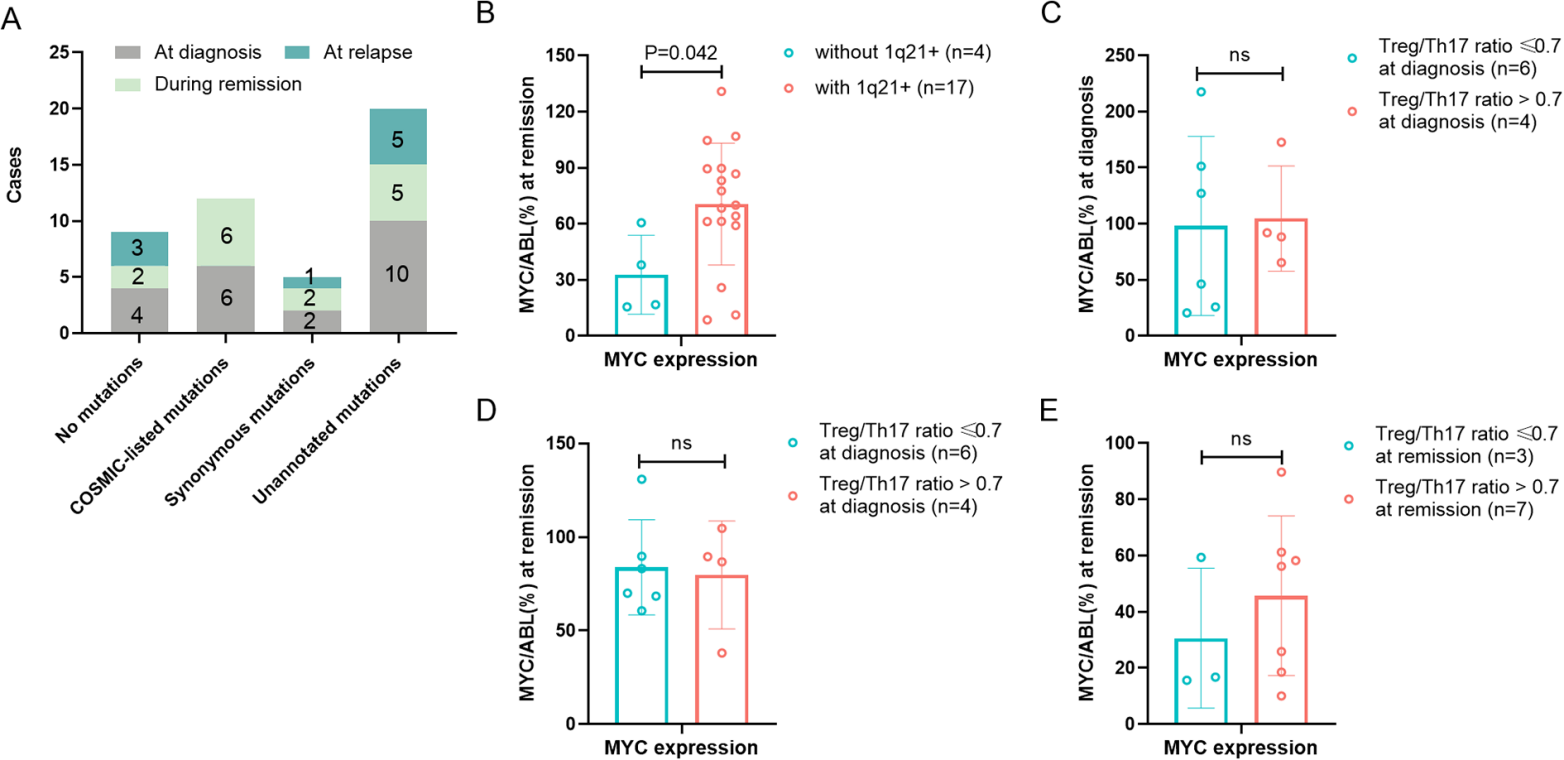
Association of *MYC* gene abnormality with 1q21 gain/amplification and Treg/Th17 Ratio. **(A)** *MYC* gene sequencing results of MM Patients at initial diagnosis, remission and relapse. The numbers marked on the graph indicate the number of patients. **(B)** *MYC* gene expression during remission in patients with or without 1q21 gain/amplification. **(C, D)** *MYC* gene expression at diagnosis and remission in high- vs low-Treg/Th17 ratio groups at diagnosis. **(E)** *MYC* gene expression at remission in high- vs low-Treg/Th17 ratio groups at remission. Treg, regulatory T cell. Th17, T helper (Th) 17 cell. *p* < 0.05 was considered statistically significant.

To comprehensively characterize *MYC* gene mutational profiles, we performed systematic sequencing analysis at three distinct clinical stages: initial diagnosis, remission, and recurrence. The complete *MYC* gene sequencing results for each disease phase are presented in **Figure 4A**. Given the lack of known hematological disease associations for any detected *MYC* mutations, we classified the sequencing results into two categories: no mutations and with mutations present (including COSMIC-listed, synonymous, and unannotated variants). At initial diagnosis, *MYC* mutations were detected in 84.6% (11/13) of patients with 1q21 gain/amplification, slightly higher than the 33.3% (1/3) observed in those without 1q21 gain/amplification (*p* =1.136), **Table 1**. Although this difference did not reach statistical significance, the observed trend suggests a potential association between 1q21 gain/amplification and *MYC* mutational status.

Quantitative analysis of *MYC* gene expression revealed that at the initial diagnosis of patients (n = 41), the median level of *MYC* gene expression was 70.55% (range: 11.60 - 2384.14%). During the remission period of patients (n = 29), the expression of the *MYC* gene was 57.57 ± 32.76%. When the disease recurred (n = 14), the expression of the *MYC* gene increased again (127.62 ± 86.72%). *MYC* gene expression varied significantly across disease stages (initial diagnosis, remission, relapse; p=0.019). Further pairwise comparisons revealed that *MYC* gene expression was significantly higher during relapse compared to remission (t=3.855, p=0.011). Quantitative analysis revealed significantly higher *MYC* expression levels in patients with 1q21 gain/amplification (70.54 ± 32.62%, n=17) compared to those without 1q21 gain/amplification (32.76 ± 21.20%, n=4) during remission (*p*=0.042), as presented in **Table 1** and **Figure 4B**. However, no significant difference in *MYC* expression was observed at initial diagnosis between patients with and without 1q21 gain/amplification (*p*=0.950, **Table 1**).

Using disease recurrence as the endpoint, ROC curve analysis identified the optimal Treg/Th17 ratio cutoff values at distinct disease stages: 1.0 (AUC = 0.750) at initial diagnosis, 0.7 (AUC = 0.583) during remission, and 1.0 (AUC = 0.671) at recurrence. At the initial diagnosis, there was no statistically significant difference in *MYC* gene expression between the group with an elevated Treg/Th17 ratio (>1.0, n = 4) and the group with a decreased Treg/Th17 ratio (≤1.0, n = 6), with expression levels of 104.64% and 98.12%, respectively (*p* = 0.888), **Figure 4C**. Likewise, there was no statistically significant difference in the *MYC* gene expression level during remission between the group with an elevated Treg/Th17 ratio (>1.0 n = 4) and the group with a decreased Treg/Th17 ratio (≤1.0, n = 6) at the initial diagnosis (79.72% vs. 83.80%, *p* = 0.819), **Figure 4D**. Patients with an elevated Treg/Th17 ratio (>0.7, n=7) during remission demonstrated *MYC* gene expression levels of 45.70 ± 28.41%, those with a reduced Treg/Th17 ratio (≤0.7, n=3) during remission showed *MYC* expression levels of 30.60 ± 24.92%. However, statistical analysis indicated no significant difference between these groups (t=0.793, *p*=0.451), **Figure 4E**.

### 3.5 Analysis of GEO and MMRF databases

Given the limited sample size in our study, we further examined the relationship between 1q21 amplification, MYC expression, FOXP3 and IL-17A using the MMRF database and GEO dataset (GSE190042). Since these genomic databases lacked direct measurements of Treg and Th17 cell frequencies, we analyzed FOXP3 (a specific Treg transcription factor) and IL-17A (the defining cytokine of Th17 cells) expression levels as surrogate markers.

The MMRF database comprised 774 CD138^+^ BM samples, including 182 (23.5%) with 1q21 gain/amplification, 586 (75.7%) without, and 6 unevaluable cases. Patients with 1q21 gain/amplification exhibited significantly higher FOXP3 expression (median: 0.18, range: 0–1.02) than those without (median: 0.16, range: 0.00–0.80; *Z*= 2.230, *p*= 0.026). In contrast, IL-17A expression did not differ significantly (*p*= 0.932), nor did the FOXP3/IL-17A ratio (*p*= 0.418). MYC expression was marginally higher in the 1q21 gain/amplification group (median: 5.78, range: 0.30–9.73 vs. 5.61, range: 0.12–9.25; *p*= 0.078). Patients were stratified into high-MYC (expression above median) and low-MYC (expression below median) groups. The high-MYC group demonstrated significantly reduced FOXP3 expression (median: 0.15, range: 0.00-1.02) compared to the low-MYC group (median: 0.19, range: 0.00-0.77; Z = 4.983, *p* =0.000). While IL-17A expression showed a modest but significant increase in the high-MYC group (median: 0.00, range: 0.00-3.47 vs. 0.00, range: 0.00-0.58; Z = 2.068, *p* = 0.039), the FOXP3/IL-17A ratio remained comparable between groups (*p* = 0.647). Analysis of the GEO dataset (GSE190042) included 93 MM patients, with 72 NDMM cases. The cohort included 48 patients with 1q21 gain/amplification and 38 patients without 1q21 gain/amplification. There were no significant differences in FOXP3, IL-17A, and MYC expression between the two groups (*p*=0.057, 0.338, and 0.114, respectively). When stratified by median MYC expression, the FOXP3/IL-17A ratio showed borderline elevation (high MYC: 1.60 ± 0.14 vs low: 1.54 ± 0.10, *p*=0.055), potentially indicating a biologically relevant trend. Notably, low expression levels of FOXP3 and IL-17A were observed, which were correlated with CD138 enrichment in the tested samples. Despite this, these findings offer valuable biological insights.

### 3.6 Treg/Th17 ratio and clinical prognosis

Among the patients available for Treg and Th17 analysis, only one patient received immunomodulator drug-based therapy and one patient received daratumumab-based therapy, respectively. The regimens are mainly classified into bortezomib-based therapy and combination therapy of bortezomib and IMiDs. There was no significant difference in the levels of Treg or Th17 between the two treatment regimens at any stage (initial diagnosis, remission, or recurrence) (**Table 2**).

**Table 2:** The Impact of treatment regimens on Treg and Th17.

Clinical indicators	Bortezomib-based therapy	Bortezomib and IMiDs	Statistical value	*p*-value
Treg at Diagnosis (%)	1.83 (0.00~9.41)	2.63 (0.00~9.30)	1.615	0.106
Treg at Remission (%)	4.25 ± 2.10	4.77 ± 2.08	0.527	0.605
Treg at Relapse (%)	4.72 ± 3.52	3.72 ± 2.37	0.673	0.509
Th17 at Diagnosis (%)	0.05 ± 0.03	0.04 ± 0.03	1.034	0.309
Th17 at Remission (%)	0.04 ± 0.03	0.04 ± 0.03	0.505	0.622
Th17 at Relapse (%)	0.04 ± 0.03	0.03 ± 0.04	0.708	0.489
Treg/Th17 at Diagnosis	0.52 (0.00~3.18)	0.66 (0.00~32.26)	0.955	0.340
Treg/Th17 at Remission	1.25 (0.59~3.29)	1.58 (0.57~6.09)	0.354	0.724
Treg/Th17 at Relapse	1.11 (0.11~3.54)	0.04 (0.00~16.20)	1.124	0.261

IMiDs, immunomodulator drugs; Treg, regulatory T cell; Th17, T helper (Th) 17 cell. *p* < 0.05 was considered statistically significant.

**Figure 5:**
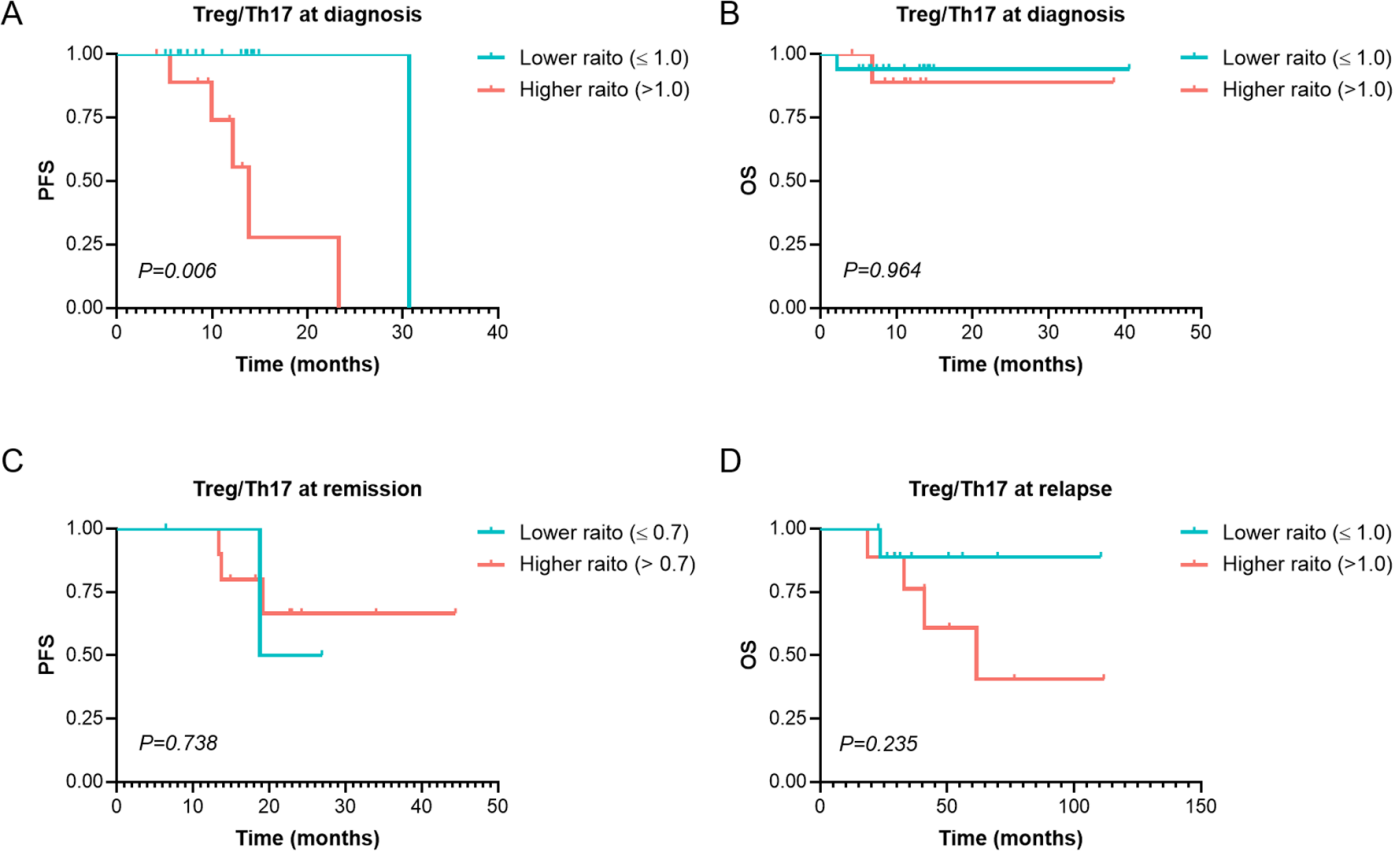
Survival analysis of PFS and OS of Treg/Th17 at Different Stages in MM Patients. **(A, B)** PFS and OS analysis in patients with Higher (>1.0) and Lower (≤ 1.0) Treg/Th17 ratio at diagnosis. **(C)** The PFS in patients with Higher (>0.7) and Lower (≤ 0.7) Treg/Th17 ratio at remission. **(D)** The OS in patients with Higher (>1.0) and Lower (≤ 1.0) Treg/Th17 ratio at relapse. PFS, Progression-Free Survival. OS, Overall Survival. MM, Multiple Myeloma. *p* < 0.05 was considered statistically significant.

A total of 34 patients were evaluated for Treg and Th17 levels at diagnosis. Analysis revealed no significant correlation between Treg or Th17 expression levels and key clinical parameters, including white blood cell count, lymphocyte-to-monocyte ratio, platelet -to- lymphocyte ratio (PLR), and LDH levels (*p* > 0.05). We evaluated the relationship between Treg or Th17 levels and optimal treatment response. The analysis revealed that the median baseline Th17 level in patients achieving a PR or better response was 3.34% (range: 0.19–10.80%), significantly higher than that in patients without PR (0.29%, range: 0–2.18%), the difference was statistically significant (Z=2.127, *p*=0.033). However, no significant differences were observed between responders (PR or better) and non-responders in Treg levels at diagnosis, the Treg/Th17 ratio at diagnosis, Th17 levels after 4–6 treatment cycles, or Th17 levels after 8–12 treatment cycles (*p* = 0.748, 0.616, 1.000, and 0.111, respectively). The median Treg/Th17 ratio at diagnosis was 0.62 (range: 0–32.26). With the Treg/Th17 cutoff value of 1.0 at initial diagnosis established by ROC curve analysis, patients were categorized into an elevated group (ratio > 1.0) and a reduced group (ratio ≤ 1.0). The PFS in the elevated group was significantly shorter (13.87 months) than that in the reduced group (30.67 months), demonstrating a statistically significant difference (χ² = 7.606, *p* = 0.006, **Figure 5A**). In contrast, the OS in the elevated group was 35.7 months, compared to 38.0 months in the reduced group, with no significant statistical difference (χ² = 0.002, *p* = 0.964, **Figure 5B**).

A total of 16 patients were assessed for Treg and Th17 levels at remission. The median Treg/Th17 ratio at remission was 1.25 (range: 0.57–6.09). Using relapse as the endpoint, ROC curve analysis determined an optimal Treg/Th17 ratio cutoff of 0.7 at remission. Patients were stratified into an elevated Treg/Th17 ratio group (ratio > 0.7) and a reduced Treg/Th17 ratio group (ratio ≤ 0.7). Analysis demonstrated that the Treg/Th17 ratio at remission had no significant effect on PFS (χ² = 0.112, *p* = 0.738; **Figure 5C**).

A total of 19 patients were evaluated for Treg and Th17 levels at relapse. The median Treg/Th17 ratio at relapse was 0.53 (range: 0–16.20). Using overall survival as the endpoint, ROC curve analysis identified an optimal Treg/Th17 ratio cutoff of 1.0 at disease relapse. Patients were stratified into an elevated Treg/Th17 ratio group (ratio > 1.0) and a reduced Treg/Th17 ratio group (ratio ≤ 1.0). Analysis revealed that the median OS in the elevated ratio group was 61.6 months, while the median OS in the reduced ratio group was not reached. However, no statistically significant difference was observed between the two groups (χ² = 1.412, *p* = 0.235; **Figure 5D**).

### 3.7 Prognosis of R2-ISS combined with Treg/Th17 ratio

**Figure 6:**
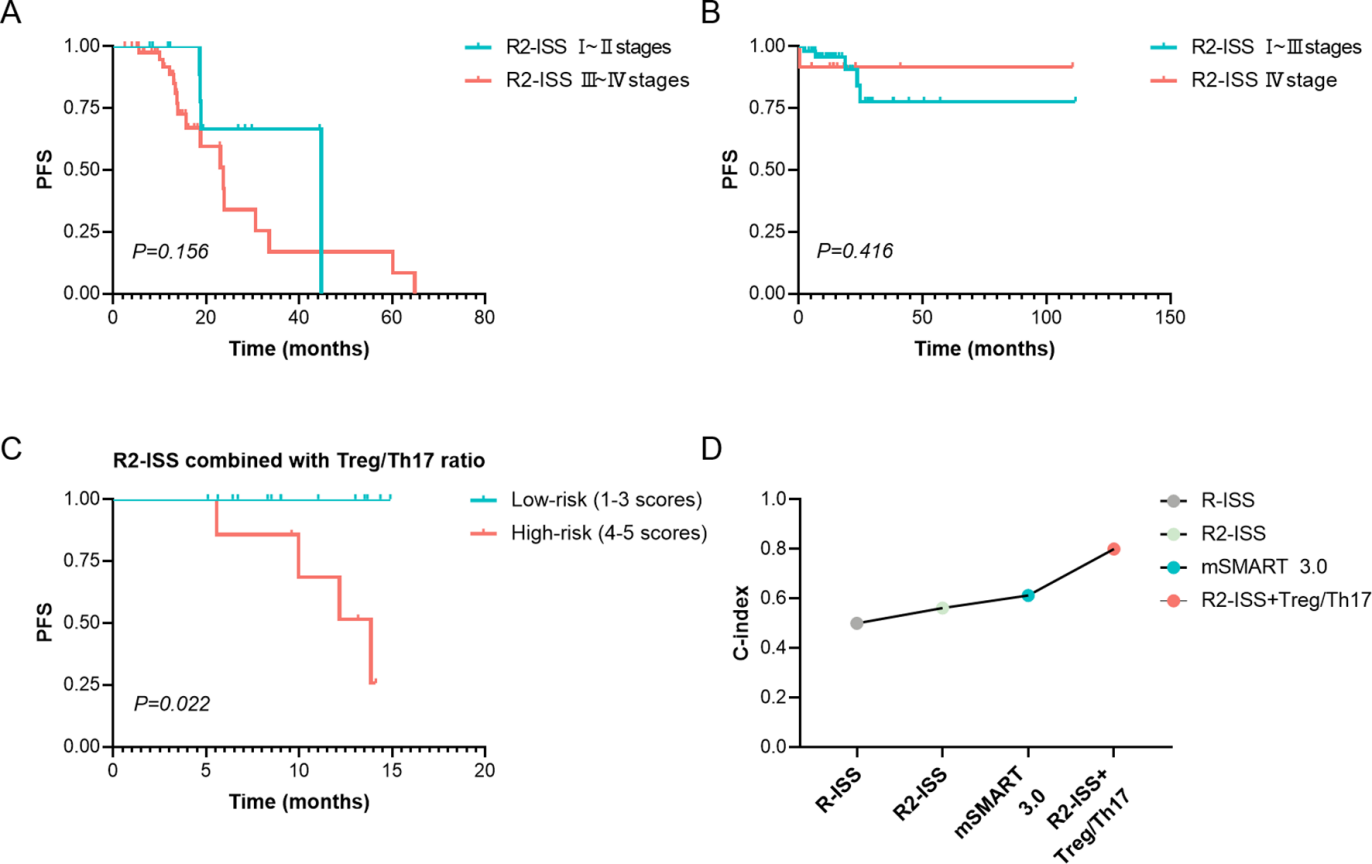
Prognosis assessment of R2-ISS or combined Treg/Th17 ratio. **(A)** The difference in PFS between R2-ISS stage I–II and stage III–IV patients. **(B)** The difference in PFS between R2-ISS stage I–III and stage IV patients. **(C)** Comparison of PFS based on the risk stratification by the combination of R2-ISS staging and Treg/Th17 Ratio at diagnosis. R2-ISS stages I, II, III and IV were assigned scores of 1, 2, 3, and 4, respectively, while Treg/Th17 ratio > 1.0 at diagnosis was assigned a score of 1. Patients with a total score of 1–3 were classified as low-risk, and those with a score of 4–5 were classified as high-risk. **(D)** C-index for the prediction of PFS by R-ISS, R2-ISS, mSMART 3.0, and the combination of R2-ISS with the baseline Treg/Th17 ratio. R2-ISS, the second revision of the International Staging System. Treg, regulatory T cell. Th17, T helper (Th) 17 cell. C-index, concordance index. PFS, Progression-Free Survival. *p* < 0.05 was considered statistically significant.

This study demonstrated that neither the Mayo mSMART 3.0 stratification nor the traditional R-ISS staging had no significant impact on PFS (*p* > 0.05). Although the median PFS was longer in patients with R2-ISS stage I or II than in patients with stage III or IV, the difference was not statistically significant (*p* > 0.05), **Table 3**. Similarly, no statistically significant difference in PFS was observed between R2-ISS stage I– II and stage III–IV patients (χ² = 2.017, *p* = 0.156, **Figure 6A**), nor between R2-ISS stage I–III and stage IV patients (χ² = 0.661, *p* = 0.416, **Figure 6B**).

**Table 3:** Univariate analysis of factors influencing Progression-Free Survival (PFS).

Clinical Indicator	Median PFS (months)	95% CI	Statistical Value	*p*-value
Gender				
male	30.67	17.36~43.97	2.694	0.101
femal	35.53	25.21~45.86		
R-ISS stage			1.322	0.516
I	18.90	18.47~19.33		
II	23.70	10.18~37.22		
III	23.90	22.60~25.20		
Mayo mSMART3.0 stratification			0.036	0.850
Standard Risk	18.77	18.60~18.93		
High Risk	23.70	11.72~35.68		
R2-ISS stage			4.247	0.236
I	35.84	26.13~45.56		
II	36.19	16.63~55.75		
III	25.16	16.40~33.91		
IV	35.50	9.45~61.56		
Platelet/Lymphocyte Ratio (>265)			0.321	0.571
No	31.43	19.62~43.25		
Yes	29.73	21.06~38.41		
Lymphocyte/Monocyte Ratio (>3.0)			0.321	0.571
No	31.43	19.62~43.25		
Yes	29.73	21.06~38.41		
Elevated LDH(≥250 U/L)			0.030	0.862
No	31.95	23.01~40.88		
Yes	27.30	18.31~36.29		
Elevated Treg/Th17 Ratio at Diagnosis(>1.0)			7.606	0.006*
No	30.67	NA		
Yes	13.87	11.22~16.51		

The median lymphocyte/monocyte ratio was 2.97 (range: 0.41–22.67), a ratio >3.0 was considered elevated, and ≤3.0 was considered reduced. The median platelet/lymphocyte ratio was 265.15 (range: 33.85–3652.78), a ratio >265 was considered elevated, and ≤265 was considered reduced. PFS, Progression-free survival. R-ISS, Revised International Staging System. R2-ISS, the second revision of the International Staging System. Treg, regulatory T cell; Th17, T helper (Th) 17 cell; LDH, lactate dehydrogenase; NA, Not applicable. *p < 0.05 was considered statistically significant.

Given that R2-ISS staging does not incorporate immune-related biomarkers, we assessed the prognostic value of combining R2-ISS staging with the Treg/Th17 ratio at diagnosis. R2-ISS stages I, II, III and IV were assigned scores of 1, 2, 3, and 4, respectively, while a baseline Treg/Th17 ratio > 1.0 was assigned a score of 1. A novel risk stratification index was developed based on the sum of the R2-ISS stage score and the baseline Treg/Th17 ratio score. Patients with a total score of 1–3 were classified as low-risk (12 cases), and those with a score of 4–5 were classified as high-risk (9 cases). Analysis demonstrated that high-risk patients, as defined by the combined R2-ISS staging and Treg/Th17 ratio at diagnosis, exhibited significantly worse PFS compared to low-risk patients (χ² = 5.211, *p* = 0.022, **Figure 6C**). The prognostic performance of the stratification models for PFS was evaluated using the concordance index (C-index). The combined R2-ISS staging and Treg/Th17 ratio at diagnosis achieved a C-index of 0.8, outperforming R-ISS staging (0.50), R2-ISS staging (0.562), and Mayo mSMART 3.0 stratification (0.61), indicating superior predictive accuracy, **Figure 6D**.

## 4 Discussion

1q21 status is not included among the high-risk markers listed by the International Myeloma Working Group’s R-ISS (34). It was not until the updated mSMART 3.0 risk stratification system in 2018 that 1q+ (gain or amplification of chromosome 1q) was formally integrated into the risk stratification criteria (35). Consequently, 1q21 has rarely been reported in most studies over the past decade. As a result, its prognostic significance within the framework of modern therapeutic approaches remains inadequately characterized. Although 1q21 gain is considered an indicator of poor prognosis (22, 40).There are literatures reporting that 1q21 has no significant impact on PFS and OS of MM patients (25, 41). Our study also demonstrated that 1q21 gain/amplification did not significantly impact PFS, which is consistent with the previous reports. The prognostic impact of 1q21 exhibits heterogeneity, which may be attributed to the presence of concurrent genetic abnormalities, advanced ISS or R-ISS stage, variations in treatment regimens, or other high-risk factors (42–45). Our study found that the proportion of patients with 1q21 gain/amplification classified as ISS stage II and R-ISS stage III was significantly higher compared to those without 1q21 gain/amplification. Additionally, the proportion of patients with 1q21 gain/amplification harboring at least one high-risk cytogenetic abnormality was significantly higher compared to those without 1q21 gain/amplification. These findings suggest that 1q21 gain/amplification may be associated with more advanced disease stages and a higher burden of concurrent high-risk genetic abnormalities. It was consistent with the above reports. Treg and Th17 cells, which are part of the CD4+ T cell subpopulation (46), play a crucial role in maintaining the balance within the immune system (47). Our study demonstrated that patients with 1q21 gain/amplification had a significantly higher Treg/Th17 ratio during remission compared to those without 1q21 gain/amplification. Intriguingly, the presence of 1q21 gain/amplification did not have a significant impact on the Treg/Th17 ratio at diagnosis. These findings suggest that although 1q21 gain/amplification does not substantially affect immune cell function at diagnosis, patients with 1q21 gain/amplification exhibited more pronounced immunosuppressive features during remission.

Limited studies have explored the regulatory role of 1q21 in Treg and Th17 cells. However, cytogenetic alterations may influence immune cell function during disease development and progression (48, 49). Integrated single-cell RNA sequencing (scRNA-seq) analysis of myeloma cells and their microenvironment revealed that gain(1q) clones correlate with expansion of specific immune cell subsets (48). The expansion of resistant clones is driven by upregulated genes for survival, proliferation, and immune evasion (48). MYC activation has been demonstrated to be closely associated with MM, and it shows a significant correlation with a shorter survival duration for patients (50, 51). Some studies have found that the combination of *MYC* rearrangement and 1q21 gain is particularly associated with a poor prognosis in MM patients (25, 52). The activation of MYC protein is dependent on MCL1 (25), which is a key gene in the 1q21 region (53). Our research revealed that during the remission phase, patients with 1q21 gain/amplification exhibited significantly higher *MYC* gene expression levels compared to those without 1q21 gain/amplification. Additionally, *MYC* mutations were more prevalent in patients with 1q21 gain/amplification (84.6%) compared to those without (33.3%). Although this difference did not reach statistical significance—likely due to limited sample size—the trend suggests a potential biological link between 1q21 gain/amplification and *MYC* mutational status. Notably, *MYC* rearrangement was detected in one patient, who also harbored 1q21 gain/amplification. Overall, patients with 1q21 gain/amplification not only showed a significantly higher Treg/Th17 ratio during the remission period, but also had a markedly increased expression of the *MYC* gene during the same disease stage.

We also observed a trend of synchronous changes between Treg/Th17 and the *MYC* gene during remission. Patients with an elevated Treg/Th17 ratio showed slightly higher *MYC* gene expression levels compared to those with a lower Treg/Th17 ratio during remission (45.70% vs. 30.60%, *p*=0.451). Although this difference was not statistically significance, the consistent trend implies a possible coordinated modulation between *MYC* expression and the Treg/Th17 ratio balance in remission. Due to the limitations of our sample size, we further validated our findings in established databases. Since these databases lacked direct measurements of Treg and Th17 cell frequencies, we assessed FOXP3 and IL-17A expression as proxies. Analysis of the MMRF cohort revealed significantly elevated FOXP3 expression but comparable IL-17A levels in patients with 1q21 gain/amplification versus non-altered cases. MYC stratification reveals differences in FOXP3 and IL-17A expression, although FOXP3/IL-17A ratios remained similar between groups. Intriguingly, the GEO cohort showed a borderline significant trend toward higher FOXP3/IL-17A ratios in MYC-high patients, potentially indicating a biologically relevant trend of MYC expression and Treg/Th17 balance, consistent with our primary findings. Previous studies (54, 55) have demonstrated that *MYC* transcriptionally upregulates PD-L1 in multiple cancer types, with direct binding to the PD-L1 promoter region driving its expression (56). Given that PD-L1 modulates the Th17/Treg balance (57, 58). Our findings suggest a potential mechanistic link between 1q21 abnormalities to Treg/Th17 imbalance via *MYC* gene overexpression. Specifically, patients with 1q21 gain/amplification may experience more robust immune suppression during remission, as evidenced by their elevated Treg/Th17 ratios and concomitant *MYC* upregulation. This immunosuppressive phenotype could plausibly contribute to an increased risk of disease relapse, highlighting the clinical relevance of 1q21 and immune imbalance.

The percentage of Th17/CD4+ T cells in PB has been shown to have good predictive value for MM (8). MA T et al. (59) reported that the percentage of Th17 cells in PB was significantly elevated in patients achieving a PR. Similarly, we observed that the proportion of Th17 cells at diagnosis was significantly higher in patients who achieved a treatment response of PR or better compared to those who did not attain remission. These findings are consistent with previous reports. Qin N et al. (60) reported that Tregs in MM patients with a high PLR was significantly higher than that in low PLR group, while Th17 cells was significantly lower than that in low PLR group. However, our study did not show a clinically significant difference in the relationship between Th17 and PLR. IMiDs have been shown to inhibit the function of CD8+T cells and Tregs (61, 62). Our study found no significant differences in Treg or Th17 cell levels between bortezomib-based therapy and combination therapy with bortezomib and immunomodulatory drugs (IMiDs). This observation may be attributed to the fact that IMiDs are often incorporated into maintenance therapy even after initial bortezomib-based induction regimens. While IMiD-containing regimens may influence Treg and Th17 cell dynamics, such effects did not translate into detectable intergroup differences in our cohort.

Several studies have documented the prognostic significance of the Treg/Th17 ratio in various other tumors (32, 63). Similarly, the equilibrium between Treg and Th17 cells also exerts a certain influence on the prognosis of multiple myeloma (MM). Anna Kulikowska de Nał ˛ecz (10) research showed that the elevated Treg/Th17 ratio was more notable at myeloma relapse. A long-term follow-up study of MM patients (64) revealed significantly higher Treg levels and lower Th17 levels in patients followed for less than 10 years compared to long-term survivors. Our study showed that patients with a higher Treg/Th17 ratio at diagnosis had a significantly shorter PFS compared to those with a lower ratio, which was consistent with the above reports. These findings suggest that long-term PFS in MM is associated with immune function, and a decrease in immunosuppression is beneficial. However, other studies have shown that the Treg/Th17 ratio is significantly lower in relapsed/refractory MM patients (65), and a reduced Treg/Th17 ratio is associated with significantly shorter OS (19). The clinical significance of changes in the Treg/Th17 ratio may vary depending on the cancer type or disease stage (66). Generally speaking, the balance between Treg and Th17 cells appears to be a critical factor in MM progression and may hold potential prognostic value.

Over the past decade, risk stratification related to MM prognosis had been continuously refined and optimized in response to the evolving needs for treatment guidance and improved outcomes in MM. In 2015, the IMWG integrated cytogenetic abnormalities and LDH levels into the ISS, proposing the R-ISS (34). Subsequently, the updated mSMART 3.0 in 2018 incorporated 1q+ into the high-risk category (35). In 2022, the European Myeloma Network proposed a new risk stratification system R2-ISS based on R-ISS (36). This system assigns scores to baseline risk factors and identifies four independent prognostic groups using a cumulative scoring approach. Notably, the R2-ISS staging system was developed and validated using extensive clinical trial data. However, its real-world application and refinement require ongoing validation and optimization. J Yan et al. (67) reported that median PFS and OS by R2-ISS differed significantly after regrouping by adjusting for R2-ISS scores. In our study, the C-index was employed to evaluate the prognostic performance of stratification indicators for PFS. The results indicated that the R2-ISS staging system combined with the Treg/Th17 ratio demonstrated superior predictive efficacy for NDMM compared to the R2-ISS alone and the mSMART 3.0 risk stratification. This indicates that the combined model integrating R2-ISS staging with the Treg/Th17 ratio at diagnosis demonstrates high predictive accuracy for disease relapse. To our knowledge, this study established the first risk prediction model incorporating immune biomarkers, demonstrating enhanced predictive capability for relapse in NDMM. This novel staging system demonstrates significant potential for predicting early relapse in NDMM, which may enable personalized therapeutic decision-making and improve clinical outcomes.

## 5 Conclusion

Our study evaluated the relationships among cytogenetic 1q21 status, *MYC* gene expression, and the immune balance between Treg and Th17 cells. Patients harboring 1q21 gain/amplification displayed a significantly higher Treg/Th17 ratio during the remission phase, accompanied by increased *MYC* gene expression levels at the same stage. This study demonstrates that 1q21 gain/amplification in MM is associated with an immunosuppressive tumor microenvironment characterized by Treg/Th17 imbalance and *MYC* overexpression during remission. The elevated Treg/Th17 ratio (>1.0) at diagnosis emerged as a powerful independent prognostic factor, significantly correlating with shorter PFS. Integration of the Treg/Th17 ratio at diagnosis into the R2-ISS staging system significantly enhances prognostic discrimination, enabling more refined risk stratification in multiple myeloma patients. These results highlight three critical clinical implications: (1) 1q21 gain/abnormalities may drive both immune dysregulation and oncogenic pathways, (2) baseline Treg/Th17 imbalance identifies high-risk patients who may benefit from intensified or immunomodulatory therapies, and (3) integration of immune biomarkers with existing risk models enhances prognostic precision in the era of MM immunotherapy. While the precise regulatory mechanisms linking 1q21 gain/amplification to Treg/Th17 imbalance remain to be fully characterized, our findings identify a potentially significant pathway worthy of further investigation. The current study’s statistical power is constrained by the small cohort size, particularly for stratified analyses of clinical subgroups. To overcome this limitation, we plan to establish multicenter collaborations based on the sample size estimated by PASS. Expanding the study population would help strengthen the statistical power and generalizability of the findings.

## Data Availability

The original contributions presented in the study are included in the article/supplementary material. Further inquiries can be directed to the corresponding author.
